# A Real-World Evaluation of Definisse Filler for Facial Rejuvenation in Indian Patients: The REDEFINE REJUVENATION Study

**DOI:** 10.7759/cureus.95475

**Published:** 2025-10-26

**Authors:** Samatha Nuthalapati, Jyotsna Deo, Karishma Balani, Rickson Pereira, Shefali Trasi Nerurkar, Indu Ballani, Shilpi Bhadani, V S Rathore, Seema Srinivasa, Chetan Y Patil, Isha Kaushik, Sanjana Dadhich, Amit Pawar

**Affiliations:** 1 Dermatology, Dr. Samatha Clinic, Hyderabad, IND; 2 Dermatology, Cutis Skin and Laser Centre, Navi Mumbai, IND; 3 Dermatology, Synova Care, Mumbai, IND; 4 Dermatology, Dr. Rickson's Dermatherapie Clinic, Mumbai, IND; 5 Dermatology, Dr. Trasi's La Piel Skin Clinic, Mumbai, IND; 6 Dermatology, BLK Max Hospital, New Delhi, IND; 7 Plastic and Reconstructive Surgery, SB Aesthetics, New Delhi, IND; 8 Plastic Surgery, Kaayakalp Clinic, Kolkata, IND; 9 Dermatology, Dr. Seema Skin Care and Laser Centre, Bengaluru, IND; 10 Medical Affairs, A. Menarini India Pvt Ltd., Mumbai, IND

**Keywords:** definisse filler, dermal filler, facial rejuvenation, hyaluronic acid filler, rheology, xtr technology, hyaluronic acid

## Abstract

Background and objective

India’s burgeoning aesthetic medicine sector has witnessed exponential growth, driven by increasing disposable incomes, social media influence, and a growing acceptance of cosmetic procedures. The approval of the first hyaluronic acid (HA) filler in 2003 initiated a wave of innovation; advancements in cross-linking and formulation now allow for tailored treatments across diverse patient needs and anatomical regions. The most recent addition to HA fillers, Definisse filler (RELIFE S.r.l., Florence, Italy) has gained attention specifically due to its proprietary manufacturing process, known as the eXcellent Three-Dimensional Reticulation (XTR) technology. However, it lacks robust real-world data in the Indian context. Hence, this study aimed to evaluate the safety and efficacy of Definisse fillers in a real-world setting among Indian patients.

Methodology

This was a multicenter, retrospective observational study that evaluated Indian adult patients who received facial rejuvenation treatments using the Definisse filler range (Touch, Restore, and Core). The study analyzed real-world clinical data to assess treatment outcomes, including efficacy and safety profiles in a diverse patient population.

Results

The study included 304 patients, with a mean follow-up duration of 3.9 months and an average age of 41.0 years. An average of 4.4 ml of Definisse filler was administered per patient. Both the Physician and Subject Global Aesthetic Improvement Scales (PGAIS and SGAIS) demonstrated immediate post-treatment improvement, with mean scores of 3.9 and 3.87, respectively. Further enhancement was observed during follow-up visits, with mean scores increasing to 4.37 and 4.33, respectively (p < 0.001 for both). Overall, about 90% of patients exhibited either 'much improvement' or 'very much improvement' on PGAIS at the follow-up visit. Most procedure-related adverse events were mild and resolved spontaneously within a few days without the need for active intervention.

Conclusions

The findings of this multicenter, real-world study indicate that Definisse fillers are clinically effective and exhibit a positive safety profile for facial rejuvenation in Indian patients. While these results show consistent clinical improvement, it must be noted that the study was designed as observational and retrospective and had a short mean follow-up. These findings provide valuable guidance for initial clinical practice, and further prospective studies are warranted to confirm long-term outcomes.

## Introduction

The quest for youthful facial aesthetics has driven significant advancements in minimally invasive cosmetic procedures, with hyaluronic acid (HA)-based dermal fillers emerging as a cornerstone of modern aesthetic medicine [1]. HA fillers are widely favored for their biocompatibility, reversible nature, and ability to restore volume, enhance contours, and reduce wrinkles by mimicking the skin’s natural hydrating properties [1-3]. They meet the demand for a biologic agent that is safe, easy to administer, effective for medically indicated applications, and associated with a low risk of allergic reactions [3-5]. Since the approval of the first HA filler in 2003, innovations in cross-linking technologies and product formulations have expanded their applications, enabling tailored treatments for diverse anatomical regions and patient needs [5-7]. 

Among HA fillers, Definisse fillers (RELIFE S.r.l., Florence, Italy) have garnered attention for their proprietary manufacturing process, which utilizes the eXcellent Three-Dimensional Reticulation (XTR) technology. The technique employs a controlled three-step manufacturing process. It involves pretreatment (temperature-controlled fracturing), crosslinking (formation of stable elastic 3D matrix), and purification (removal of free HA and unwanted crosslinking agents) [8]. This technology enables the production of a product with a stable three-dimensional HA matrix, yielding consistent viscoelastic properties, increased G′, and low extrusion force [8-10]. These rheological properties are believed to contribute to enhanced product stability and sustained volume correction in mid-to-deep dermal volumization [8].

While randomized controlled trials (RCTs) provide important evidence on efficacy and safety in idealized settings, real-world studies are necessary to confirm these outcomes in diverse patient populations and clinical practices [11]. Crucially, there is a distinct gap in robust, large-scale, real-world data regarding the clinical use and outcomes of the Definisse range of HA fillers, specifically within the Indian patient population. Hence, this REDEFINE REJUVENATION study aims to describe the efficacy, safety, and patient satisfaction associated with the Definisse range (including Definisse Core, Definisse Restore, and Definisse Touch) across a broad spectrum of facial rejuvenation applications in this setting.

## Materials and methods

The REDEFINE REJUVENATION study aimed to assess the real-world efficacy and safety of Definisse HA fillers (Core, Restore, and Touch) for facial rejuvenation in an Indian patient cohort. This multicenter, retrospective, observational study was conducted across nine centers in India, all staffed by injectors with over 10 years of experience in filler procedures. Patients included in the study underwent facial contouring procedures with Definisse fillers based on the treating physician’s clinical judgment between September 2023 and January 2025 and had completed at least one post-procedure follow-up. Patient demographics, including age and gender, were documented along with treatment specifics, including the type and quantity of Definisse fillers used and any adverse effects.

The aesthetic outcome was evaluated using the 5-point Global Aesthetic Improvement Scale (GAIS) reported by the physician and subject (Physician Global Aesthetic Improvement scale-PGAIS, and Subject Global Aesthetic Improvement scale-SGAIS) [12-13]. On this scale, a score of 1 indicated a 'worse' outcome, where the appearance had deteriorated compared to the baseline condition. A score of 2 signified 'no change' from the pre-treatment state. A score of 3 corresponded to an 'improved' appearance, defined as a clear enhancement that would still benefit from touch-up treatment. A rating of 4 represented a 'much improved' state, characterized by a marked aesthetic improvement that was not yet optimal. Finally, a score of 5 was assigned for a 'very much improved' outcome, representing the achievement of an optimal aesthetic result [12-13].

A comprehensive facial assessment was performed, and an individualized treatment plan was developed, targeting one or multiple facial areas to achieve holistic results. Pre-treatment included application of a topical anaesthetic for 20 minutes to reduce discomfort during the procedure. Depending on the indication, either a needle or a cannula was used to provide the best result and minimize vascular complications. Post-treatment care included instructions to apply anti-infective ointment for seven days and to avoid touching or massaging the treated area for 48 hours. Patients were also asked to avoid smoking, alcohol, strenuous exercise, and exposure to direct sunlight.

GraphPad Prism (version 9) software was used for data analysis. The results were presented in the form of descriptive statistics. Frequency and percentages were used to analyze categorical data, while mean and standard deviation (SD) were used to summarize continuous data. Non-parametric paired data were analyzed using a Wilcoxon Signed-Rank Test, and a p-value < 0.05 was considered statistically significant for all comparisons. Being a retrospective, real-world observational study that analyzed all consecutive patients who met the selection criteria during the study period, a formal sample size calculation was not undertaken.

## Results

Three hundred and four patients from nine centres with ages ranging from 21 to 80 years (mean age: 41.0 years) were considered for analysis. The duration of the patients’ final follow-up visits ranged from two weeks to eight months, with an average visit time of 3.9 months. A larger proportion of participants were female (84.2%) compared to males (15.8%), reflecting the growing trend and greater acceptance of aesthetic treatments among women. The average amount of Definisse filler used per patient is presented in Table 1.

**Table 1:** Quantity of filler used per patient

Definisse filler	Average ml per patient
Touch	1
Restore	1.8
Core	1.6

Higher average volumes of Restore and Core used per patient compared to Touch strongly indicate that correcting underlying facial volume deficit is the predominant treatment goal for patients in this setting. The mid-face and lower face were the most treated areas (Table 2), of which the tear trough (24.86%) was the most common (Table 3), likely reflecting the fact that sunken under-eyes and general mid/lower face volume deficits are the most prominent aesthetic concerns prompting patients to seek treatment. Concomitant procedures at the follow-up visit were done in 40 (13.16%) patients. Procedures included thread lift, platelet-rich plasma (PRP), toxins, chemical peels, high-intensity focused ultrasound (HIFU), skin boosters, exosomes, facial microneedling, and radiofrequency (MNRF).

**Table 2:** Parts of face treated

Part of the face	No. of indication	Percentage of total indication
Upper face	41	7.56
Mid face	241	44.46
Lower face	260	47.97
Total	542	100

**Table 3:** Common indications of face treated

Common indication (>5%)	No. of indication	Percentage of total indication
Tear troughs	135	24.86
Chin	104	19.15
Cheeks	62	11.42
Nasolabial folds	57	10.50
Lips	51	9.39
Temples	41	7.55

**Figure 1:**
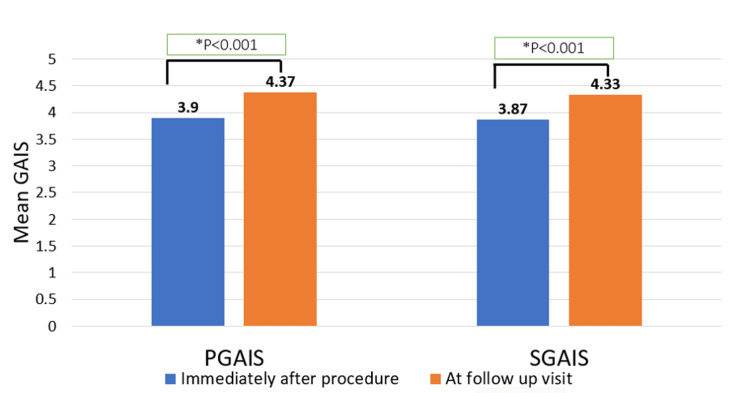
Mean GAIS rating immediately after the procedure and at the follow-up visit ^*^P-value using the Wilcoxon Signed-Rank test. For PGAIS, p < 0.001 (Z = 9.3, r = 0.7). For SGAIS, p < 0.001 (Z = 9.7, r = 0.7) GAIS: Global Aesthetic Improvement Scale; PGAIS: Physician Global Aesthetic Improvement Scale; SGAIS: Subject Global Aesthetic Improvement Scale

The mean PGAIS and SGAIS scores immediately after treatment were 3.9 ± 0.84 and 3.87 ± 0.87, respectively. These improved significantly during the follow-up visits (4.37 ± 0.66 and 4.33 ± 0.69, respectively, p < 0.001) (Figure 1). Furthermore, the majority of patients reported immediate treatment outcomes as “3” and above; that is, appearance improved to "very much improved" compared to pre-treatment in terms of PGAIS and SGAIS.

**Figure 2:**
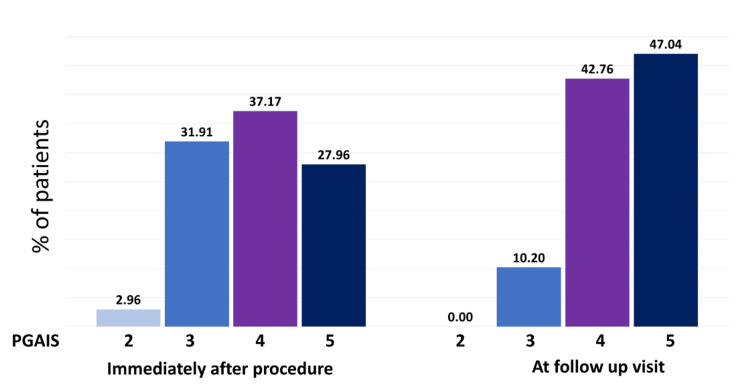
Proportion of patients showing improvement on PGAIS rating immediately after the procedure and at the follow-up visit PGAIS: Physician Global Aesthetic Improvement Scale

**Figure 3:**
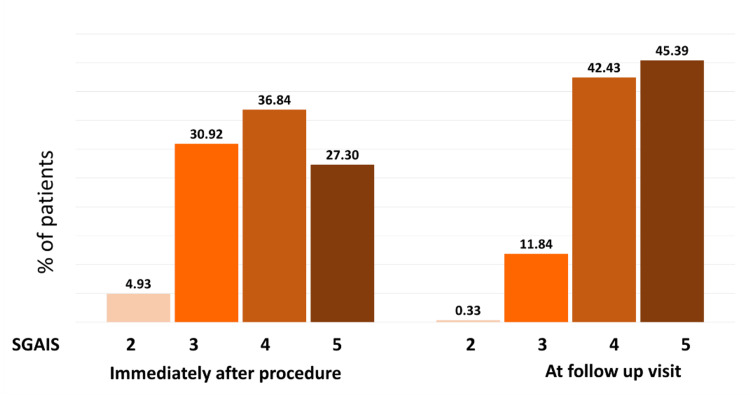
Proportion of patients showing improvement on SGAIS rating immediately after the procedure and at the follow-up visit SGAIS: Subject Global Aesthetic Improvement Scale

**Figure 4:**
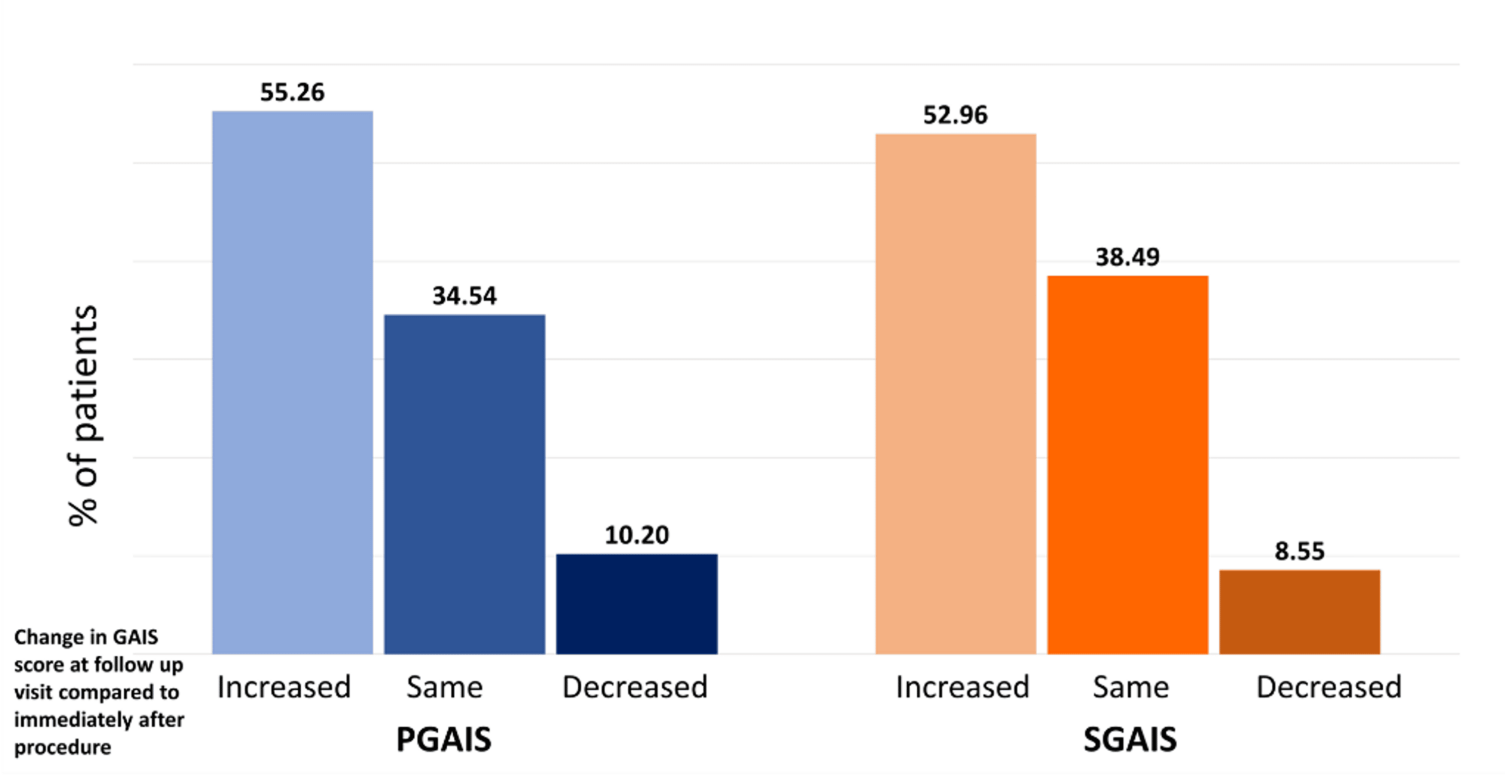
Change in GAIS score at follow-up visit compared to immediately after the procedure PGAIS: Physician Global Aesthetic Improvement Scale; SGAIS: Subject Global Aesthetic Improvement Scale

**Figure 5:**
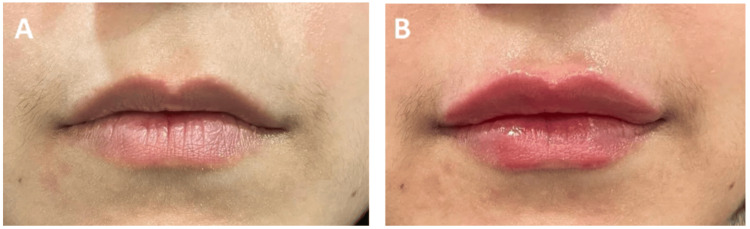
Clinical photographs of a patient who underwent filler procedure - 1 A 29-year-old female who underwent lip fillers using 1 ml of Definisse Touch: A) Pre-procedure. B) Immediately post-procedure

**Figure 6:**
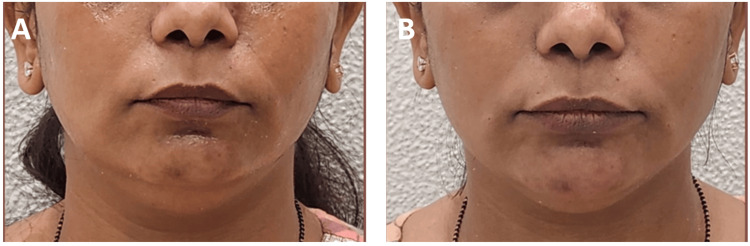
Clinical photographs of a patient who underwent filler procedure - 2 A 42-year-old female who underwent lower-face filler using 2 ml of Definisse Core and 2 ml of Definisse Restore: A) Pre-procedure. B) Immediately post-procedure

**Figure 7:**
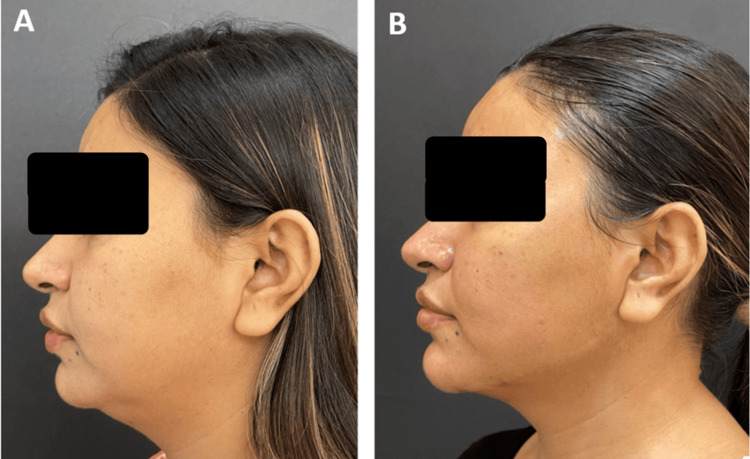
Clinical photographs of a patient who underwent filler procedure - 3 A 29-year-old female who underwent mid- and lower-face fillers using 4 ml of Definisse Core and 1 ml of Definisse Restore: A) Pre-procedure. B) Post-procedure after 1 month

**Figure 8:**
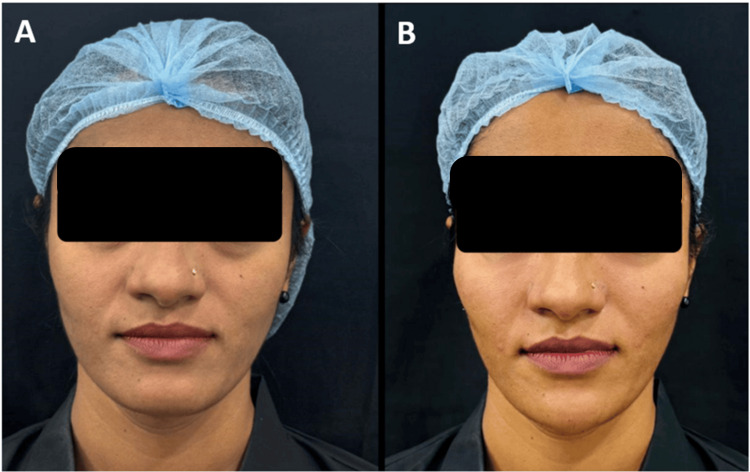
Clinical photographs of a patient who underwent filler procedure - 4 A 26-year-old female who underwent mid- and lower-face fillers using 2 ml of Definisse Core and 1 ml of Definisse Touch: A) Pre-procedure. B) Post-procedure after 1 month

**Figure 9:**
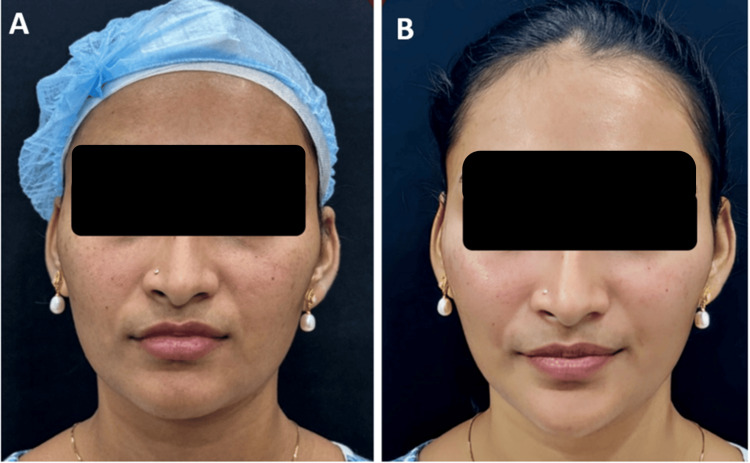
Clinical photographs of a patient who underwent filler procedure - 5 A 23-year-old female who underwent mid and lower face fillers using 1 ml of Definisse Core and 1 ml of Definisse Touch: A) Pre-procedure. B) Immediately post-procedure

At the follow-up appointment, 47.04% of patients and 45.39% of physicians reported the treatment outcome as "5," which indicates "marked improvement" in appearance, when compared with immediate PGAIS and SGAIS scores (Figures 2-3). Subsequent analysis revealed that about half of the study population had a 1-point increase in their GAIS score at the follow-up appointment compared to immediately post-procedure (Figure 4). Furthermore, only about 10% of patients showed a decrease in GAIS score (either from 5 to 4, or 4 to 3, or 3 to 2); however, none demonstrated any worsening of their condition (GAIS score 1) at follow-up. Adverse events were predominantly mild (e.g., transient erythema, injection site pain, swelling, and bruising) (Table 4), and most of them resolved spontaneously without active intervention within seven days. No severe adverse events were reported. Figures 5-9 display clinical photos of patients taken both prior to and post Definisse fillers treatment.

**Table 4:** Adverse reactions

Adverse reactions	Number of patients (n)	Percentage (%)
Injection site pain	208	68.4
Erythema	190	62.5
Localised edema	170	55.9
Bruising	85	28.0
Headache	9	3.0

## Discussion

In the last decade, injectable fillers have been used in order to counterbalance aging effects by replacing lost volume and providing structural support to the midface area [14,15]. In particular, HA fillers have emerged as the leading agents in aesthetic medicine procedures. Moreover, several studies have demonstrated HA's potential due to its safety, versatility, efficacy, and low risk of inducing allergic reactions [16,17].

Definisse fillers, developed using the XTR technology, constitute a class of HA-based dermal fillers designed for enhanced stability, longevity, and clinical efficacy [8,15,18]. Definisse Core is designed for structural volumizing in areas requiring projection (e.g., jawline, chin). Its high G’ of 426.76 resists soft tissue compression, maintaining shape under dynamic forces. Definisse Restore is suited for midface restoration and contouring with a G’ of 292.37; it balances elasticity and spreadability for better results. Definisse Touch targets the tear trough area, lips, and fine lines; the low G’ of 153.58 allows easy blending into the dermis, reducing visibility of wrinkles [8,18,19]. The current study demonstrated clinician- and patient-reported improvements in aesthetic outcomes, sustained over the follow-up period. Adverse events were mostly mild and resolved without active intervention, aligning with the safety profiles reported in prior hyaluronic acid filler studies, including that of Definisse fillers [18,20].

A study by Muti on midface restoration by high G’ filler with XTR Technology found it to be safer and effective [20]. A recently published prospective 18-month study evaluated the long-term tolerability and clinical efficacy of Definisse Core in providing volumizing effects [21]. According to the investigator’s GAIS, 76% of subjects were rated as “much improved” to “very much improved” immediately after the initial treatment. This improvement was sustained for six months and was observed in 74% of participants. By 18 months, it had declined in up to 19% of subjects, yet the overall enhanced outcome remained, with 94% of participants retaining improvement from the initial 100% following the first injection. Moreover, 62.0% of subjects rated themselves as “much improved” or “very much improved” after the initial treatment and at 18 months, indicating sustained long-term improvement. The improvement in facial volume loss was also notable over the 18-month observation period, increasing from 74% of subjects as assessed by the investigator to 89% by the end of the study. The analysis demonstrated sustained midface volumization, enhanced skin quality, and increased skin thickness for up to 18 months post-injection. The study also reported high satisfaction ratings from both patients and injectors, with all adverse reactions managed effectively [21]. This is believed to result from the distinct rheological properties of Definisse Core compared to other monophasic fillers [15,21].

Definisse Core demonstrates the highest elasticity and resistance to deformation, with a G' value of 426.76 Pa (at 0.7 Hz). Classified as a volumizer, it has the highest G' among other volumizer fillers in its class, highlighting its better performance for facial volume restoration [8,15]. Sustained volume correction seen with Definisse Core is tentatively attributed to the exposure of water-binding sites post-cross-link degradation. The HA filler-mediated tissue regeneration and remodeling remain nascent concepts and therefore warrant additional research to explore the molecular basis of the tissue response [8,15,21]. Moreover, among monophasic fillers, Definisse Core and Restore stand out for their higher degree of crosslinking (CrD%). The degree of crosslinking is considered to directly correlate with a filler's mechanical strength and longevity. Notably, Definisse Core has the highest level of CrD% at 5%, which provides it with longevity, making it a preferred choice for application in the deeper planes of the face [8,15].

Definisse filler has emerged as a pragmatic option for diverse groups of patients, including those previously excluded from trials. Its favorable safety profile supports its use in clinics with varying resource levels, while high satisfaction rates suggest meaningful impacts on patient-reported outcomes [8]. This is the first data analysis conducted among Indian patients and the largest real-world study to date using Definisse fillers. However, it also has a few limitations, such as its retrospective design, lack of a control group, and a short mean follow-up of about four months. Also, variability in injector techniques across centers is an inherent limitation of this retrospective, real-world design. Our findings need to be validated via larger prospective studies with longer follow-ups.

## Conclusions

This multicentre, real-world study provides foundational data indicating that Definisse fillers are clinically beneficial and exhibit a favorable safety profile for facial rejuvenation in the Indian patient population. The consistent performance observed supports the potential advantages of the unique XTR technology across varied clinical settings, offering practitioners a promising tool for personalized aesthetic enhancement and to successfully guide initial clinical practice. However, given the study’s limitations related to its retrospective and observational design, along with the relatively short mean follow-up, further prospective controlled studies are recommended to analyze long-term outcomes.
